# Synthetic Procedures Leading towards Aminobisphosphonates

**DOI:** 10.3390/molecules21111474

**Published:** 2016-11-04

**Authors:** Ewa Chmielewska, Paweł Kafarski

**Affiliations:** Department of Bioorganic Chemistry, Faculty of Chemistry, Wrocław University of Science and Technology, Wrocław 50-370, Poland; pawel.kafarski@pwr.edu.pl

**Keywords:** bisphosphonates, bisphosphonylation procedures, Vilsmayer-Haack-like reactions, functionalization of aminoalkylbisphosphonates

## Abstract

Growing interest in the biological activity of aminobisphosphonates has stimulated the development of methods for their synthesis. Although several general procedures were previously elaborated to reach this goal, aminobisphosphonate chemistry is still developing quite substantially. Thus, innovative modifications of the existing commonly used reactions, as well as development of new procedures, are presented in this review, concentrating on recent achievements. Additionally, selected examples of aminobisphosphonate derivatization illustrate their usefulness for obtaining new diagnostic and therapeutic agents.

## 1. Introduction

Bisphosphonates are a class of compounds that are currently receiving significant attention. Over 50 new papers are seen each week when searching the literature with the keyword “bisphosphonate” via Web of Science. More than 17,000 various bisphosphonate structures have been synthesized and described in the literature [1]. Most papers concern their important subclass, namely aminobisphosphonates. This strong interest results from these compounds acting as strong inhibitors of bone resorption, with several representatives of this class already commercialized as drugs of choice for the treatment of osteoporosis, skeletal complications of malignancy, Paget’s disease, multiple myeloma, hypercalcemia and fibrous dysplasia [2,3,4,5,6,7] Consequently, most of the papers are devoted to various clinical aspects of the anti-resorptive effects that bisphosphonates exert towards bone tissues; however, there is also a growing interest in their applications as anticancer and antibacterial agents [5,6,7]. Additionally, aminobisphosphonic acids have found important industrial applications, largely as inhibitors of scale formation and as corrosion inhibitors, actions which result from their ability to complex metal ions [8,9,10].

Thus, simple and effective procedures for their synthesis are becoming increasingly important. However, only a few general reactions leading to these compounds have been described to date and are only partially reviewed in the literature [11,12]. Novel reports are mostly concentrated on modifications and improvement of these procedures, and there are only a few papers aiming at new reactions, which results from the commonly applied procedures being simple, economical and effective.

In this paper, we comprehensively review the recent studies (supplemented by older papers if necessary) on reactions applied to synthesize the most important class of bisphosphonates—aminobisphosphonates—and discuss their scope and limitations.

## 2. Overview of Synthetic Procedures

There are contradictory reports of the origin of bisphosphonates [13]. Most likely, the first one was obtained in the 19th century by Nikolay Menschutkin and/or Theodor Saltzer as an impurity in reactions designed to obtain different compounds. It was further identified by Hans von Baeyer and Wilhelm Heideprim as 1-hydroxyethanebisphosphonic acid.

There are only a few general methods for the synthesis of aminobisphosphonates. However, there are many individual procedures described for their preparation [11,12]. In this review, the following general reactions are presented: (i) starting from carboxylic acids and (ii) their amides; (iii) reactions using nitriles and (iv) isonitriles as substrates; (v) syntheses based on addition of phosphites to oxophosphonates; (vi) three-component condensation of amines, trialkyl orthoformates and dialkyl phosphites; (vii) addition of amines to vinylidenebisphosphonates; and (viii) functionalization of simple bisphosphonates treated as building blocks for the preparation of more complex structures. Additionally, some specific and non-conventional procedures that have been elucidated will be presented in this review.

### 2.1. Synthesis from Carboxylic Acids

**Scheme 1:**
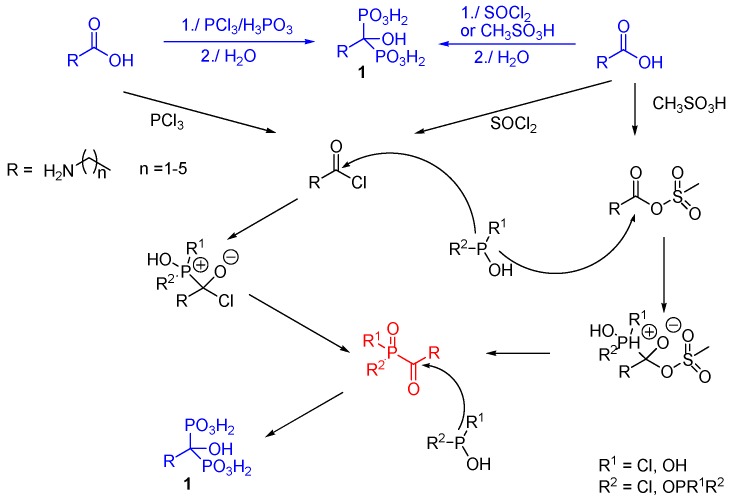
Presumable mechanism of the synthesis of 1-hydroxy-1,1-bisphosphonic acids.

1-Hydroxyethylidene-1,1-bisphosphonic acids are perhaps the oldest group of bisphosphonates. They are standardly prepared by a large-scale, one-step reaction of carboxylic acids with phosphorus trichloride and phosphorous or phosphoric acids, followed by hydrolysis with water; the procedure was optimized by Kieczykowski et al. [14]. The reaction is carried out in selected solvents (phenylsulphonic acid, various phenols, chlorobenzene, diphenyl ether or ionic liquids) with sulfone and methanesulphonic acid being preferred choices [11,15,16,17,18,19,20]. Despite many theories [19,21,22,23], the exact mechanism of this reaction is not fully understood; however, the formation of acid chloride as a first intermediate has been undoubtedly demonstrated (Scheme 1). This intermediate may react with methanesulphonic acid (when used as a solvent), and the formed mixed anhydride is also considered a potential intermediate for the next step [24], which is an Arbuzov-like reaction of phosphorus acid or one of its several derivatives (including anhydrides of variable structure [17]) formed during the reaction. The formed derivative of ketophosphonate is a substrate for the addition reaction of trivalent P-OH species, and bisphosphonate is obtained (Scheme 1). It has also been documented that the use of phosphorous acid could be omitted if water was added to the reaction medium, and thus this compound was formed in situ.

**Scheme 2:**
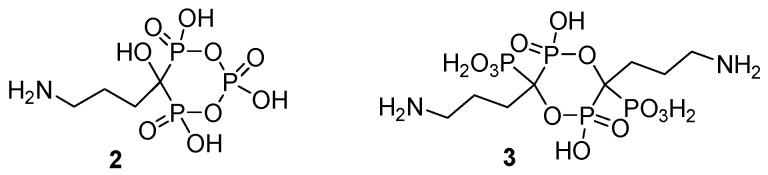
Cyclic products of the reaction between carboxylic acids and phosphorous acid in methanesulphonic acid.

This reaction was commonly used for the preparation of a wide variety of anti-osteoporotic bisphosphonic acids containing free amino groups (so called dronic acids, compound **1**). In this case, free unblocked amino acids are used as substrates, and the final neutralization of reaction mixtures to a pH of approximately 4 causes precipitation of the desired products, which are formed in satisfactory yields and are of good purity [16,23,24,25,26]. Because of its technological importance, this reaction is still quite intensively studied and optimized; however, most of the studies are done in industrial laboratories, and their results are mostly disseminated as patents. Consequently, it is difficult, if not impossible, to determine which conditions are optimal for the synthesis of individual drugs. It is important because, as in the case of any multicomponent reaction, a synthetic course is strongly dependent on applied conditions and molar ratios of reagents. For example, one of the patents reports that the omission of phosphorus chloride in the reaction of 4-aminobutyric acid as the substrate in methanesulphonic acid provides mixtures of anhydrides as final products (compounds **2** and **3**, Scheme 2) [11]. This corresponds well to the known tendency of phosphonic acids to cyclize.

Among modifications of this procedure are (i) replacement of phosphorus trichloride with thionyl chloride to generate acid chloride in the first reaction step [11]; (ii) replacement of acid by its chloride or anhydride [11] or (iii) t-butyl ester [27]; (iv) blocking the amino moiety in the case of α-amino acids [28]; and (v) application of microwave-assisted procedures [29].

### 2.2. Synthesis from Amides

**Scheme 3:**
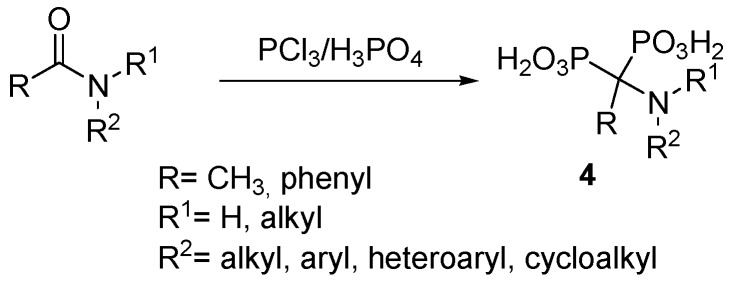
Bisphosphonylation of amides.

Although studies on the biological activity of 1-amino-1,1-bisphosphonic acids are scarce, a significant number of procedures for their preparation have been described. They have recently been reviewed by Romanenko and Kukhar [12]. Amides, being highly stable and easily available compounds, are substrates of choice, and the most commonly applied procedures are simple modifications of those elaborated for carboxylic acids. The most straightforward are reactions of *N*-acylamines and *N*-formylamines with phosphorus trichloride and phosphorous acid [30,31,32], phosphorus tribromide [33], or triphosgene [34], which provide a wide structural variety of 1-amino-1,1-bisphosphonic acids (compound **4**, Scheme 3). They are based on modification of the first procedure elaborated by Plöger et al. with formamide as a substrate [35].

**Scheme 4:**
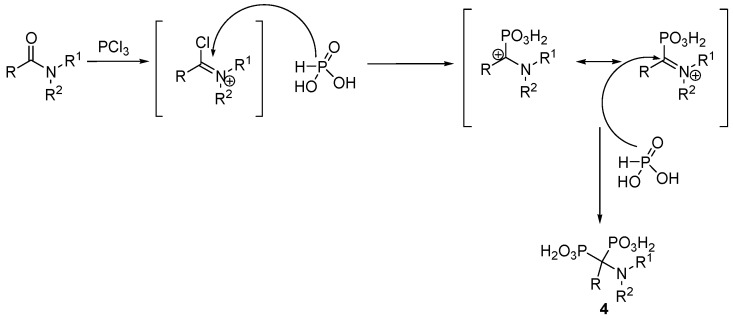
Bisphosphonylation of amides and the presumable mechanism of this reaction.

The exact reaction mechanism in this case is also not fully known; however, a Vilsmayer-Haack-like route via iminium ion is postulated here (Scheme 4).

**Scheme 5:**
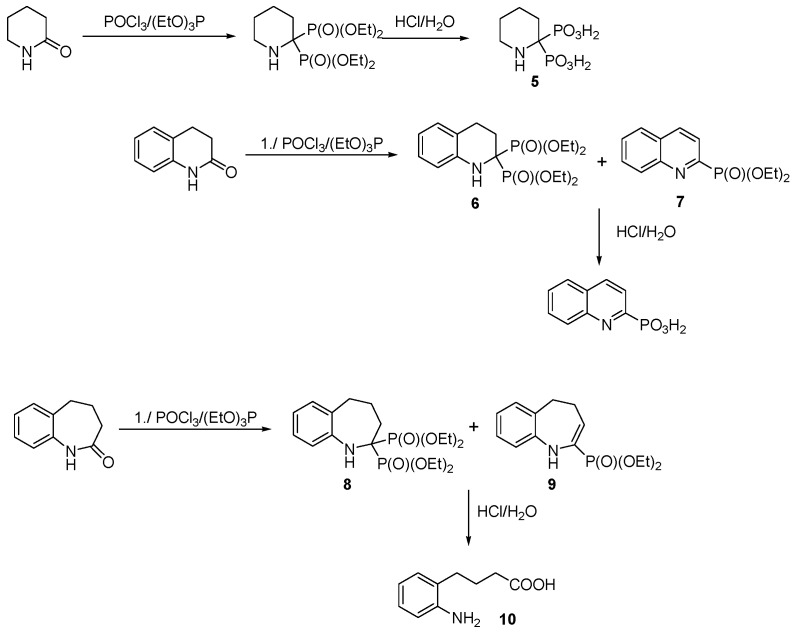
Representative reactions of lactams with triethyl phosphite prompted by phosphoryl chloride.

Another possibility is using trialkyl or trimethylsilyl phosphites in reactions prompted by phosphoryl chloride [36,37,38,39], trifluoromethanesulphonic anhydride (Tf_2_O) [40], zinc chloride [41] or trimethylsilyl trifluoromethanesulfonate [42,43]. Unfortunately, primary amides are not suitable substrates for this reaction [38]. The use of triethyl phosphite and phosphoryl chloride appeared to be especially useful when lactams were used as substrates, resulting in high yields of aminomethylene-*gem*-bisphosphonates (Scheme 5) [36,37,38,39]. They were readily hydrolyzed, yielding corresponding bisphosphonic acids (representative example compound **5**). On the other hand, this reaction is not suitable for the conversion of benzoannulated lactams, and mixtures of the desired bisphosphonates and monophosphonates of variable structures were obtained (compounds **6**, **7**, **8** and **9**, Scheme 5). In fact, monophosphonates are usually major products, and the reaction course is dependent on the size of the substrate aliphatic ring [44]. Additionally, these bisphosphonates and monophosphonates appeared to be unstable upon acid hydrolysis and upon storage, and undergo degradation with cleavage of the carbon-to-phosphorus bond (for example compound **10**, Scheme 5). Thus, corresponding acids have not yet been obtained.

**Scheme 6:**
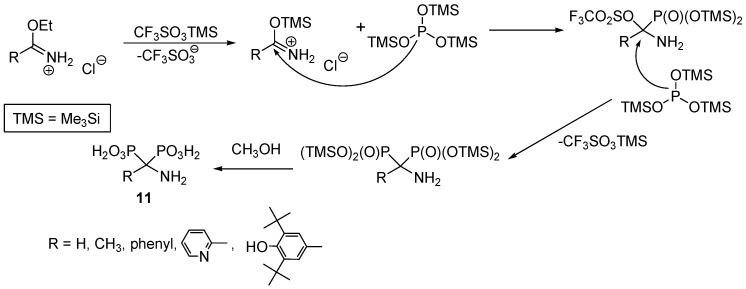
Imine salts as substrates for preparation of bisphosphonic acids.

The formation of a Vilsmayer-Haack-like intermediate is also proposed in this case with the further addition of a nucleophilic phosphorus reagent. This assumption is additionally supported by studies on the use of structurally variable imine salts as substrates for synthesis of compounds **11** (Scheme 6) [41,42,43,44,45].

**Scheme 7:**
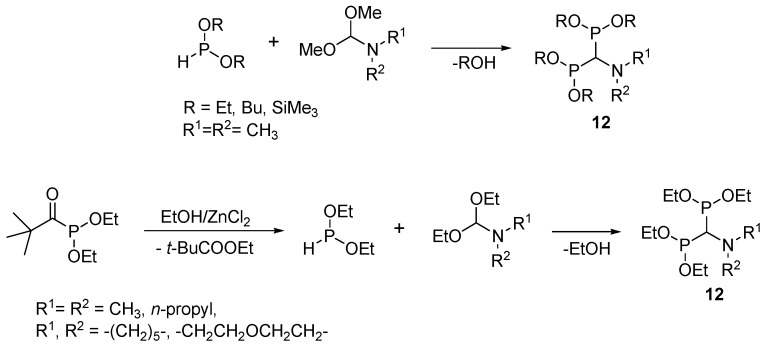
Reaction of diethoxyphosphine with the acetal of dimethylformamide.

The reaction of dibutoxyphosphine or bis(trimethylsiloxy)phosphine with dimethylformamides in the presence of trimethylsilyl trifluoromethanesulfonate affords the corresponding aminomethylenebisphosphonites **12** in high yields (Scheme 7) [41,42,43]. Similar products were obtained by reacting diethyl pivaloylphosphonite with dialkylformamides in the presence of excess ethanol and catalytic amounts of zinc chloride (Scheme 7). Pivaloylphosphonite decomposes in these reaction conditions, yielding diethoxyphosphine, which is the real substrate of the reaction [41].

**Scheme 8:**
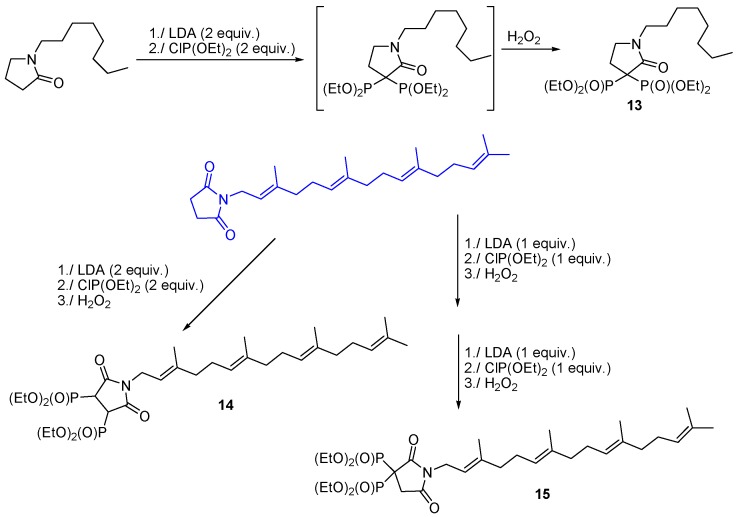
Synthesis of bisphosphonate lactams.

*N*-Octylpyrrolidinone treated with LDA, followed by the addition of diethyl phosphorochloridite and oxidation of the reaction mixture with 30% hydrogen peroxide resulted in bisphosphonylated lactam **13** with excellent yield (Scheme 8). This reaction was then applied for the synthesis of *N*-geranylated lactams **14** and **15** and linear amides, potential inhibitors of farnesyl:protein transferase [46]. In the case of cyclic imides, in which two equivalent positions for enolate formation are present on the imide rings, and thus two carbon atoms may be phosphonylated, the structure of the product was dependent on the mode of reaction. If phosphonylation was carried out in one step, two carbon atoms were phosphonylated and vicinal bisphosphonate was obtained. Step-by-step reaction resulted in the predomination of the phosphonylation of one carbon atom, and the desired gem-bisphophonate was the major product. 

### 2.3. Synthesis from Nitriles

**Scheme 9:**
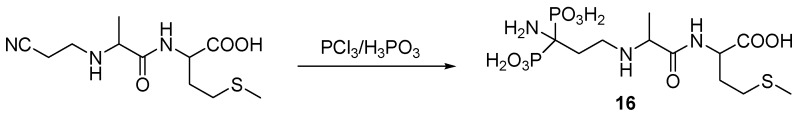
Representative example of the synthesis of peptidylbisphosphonates from nitriles.

1-Aminoalkylidene-1,1-bisphosphonic acids could also be prepared from nitriles by applying the same procedures as in syntheses starting from amides. Despite being readily available substrates, there is limited literature on their use for that purpose. The most popular route is the reaction of nitriles with phosphorous acid, in some cases prompted by phosphorus trichloride and phenylsulphonic or methylsulphonic acids [12,31,47,48]. The conditions of this reaction are sufficiently delicate to use peptidyl nitriles as substrates, which was demonstrated using cyanoethyl derivatives of dipeptides (for example, peptide **16**, Scheme 9) [49].

**Scheme 10:**
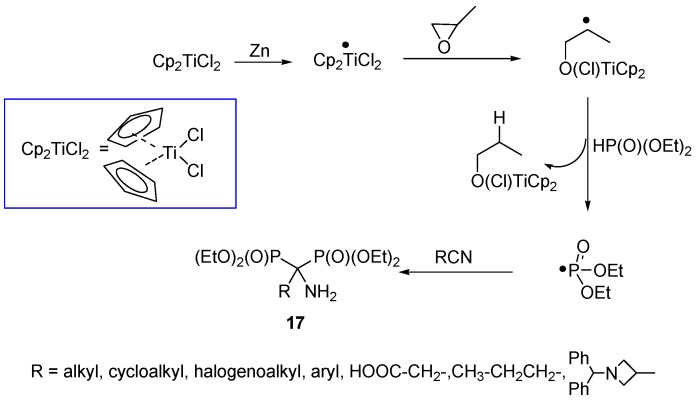
Radical procedure for the synthesis of bisphosphonates from nitriles.

Recently, elegant, mild, and atom economical double phosphonylation of nitriles in the presence of titanocene has been described. This procedure used induced phosphorus-centered radicals mediated by titanocene dichloride (Cp_2_TiCl_2_) (Scheme 10) and provided a wide structural variety of aminobisphosphonates **17** [50]. This is a double radical transfer reaction initiated by the reaction of titanocene with zinc dust and the transfer of the obtained radical to epoxypropane by cleavage of its oxirane ring, followed by transfer of this radical to diethyl phosphite, which in turn reacts with nitrile (Scheme 10). The reaction carried out without epoxide, under microwave stimulation, also results in the desired bisphosphonates, although it is accompanied by formation of many side products.

### 2.4. Synthesis from Isonitriles

**Scheme 11:**
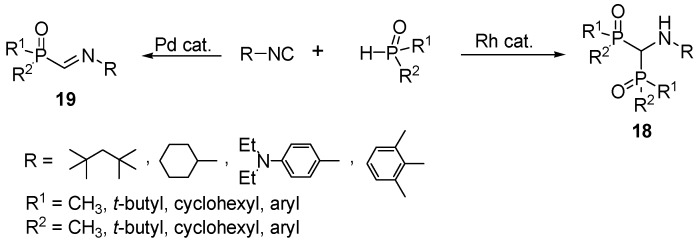
Addition of *H*-phosphine oxides to isonitriles catalyzed by metallocatalysts.

Isonitriles, which are quite common substrates, especially in multicomponent reactions [51], have been seldom used for the preparation of aminobisphosphonates **18**. Their reactions with *H*-phosphine oxides carried out in the presence of typical palladium catalysts (Pd_2_dba_3_) afforded the product **19** of monophosphonylation, whereas the use of various rhodium catalysts afforded products of diphosphonylation (Scheme 11) [52].

**Scheme 12:**
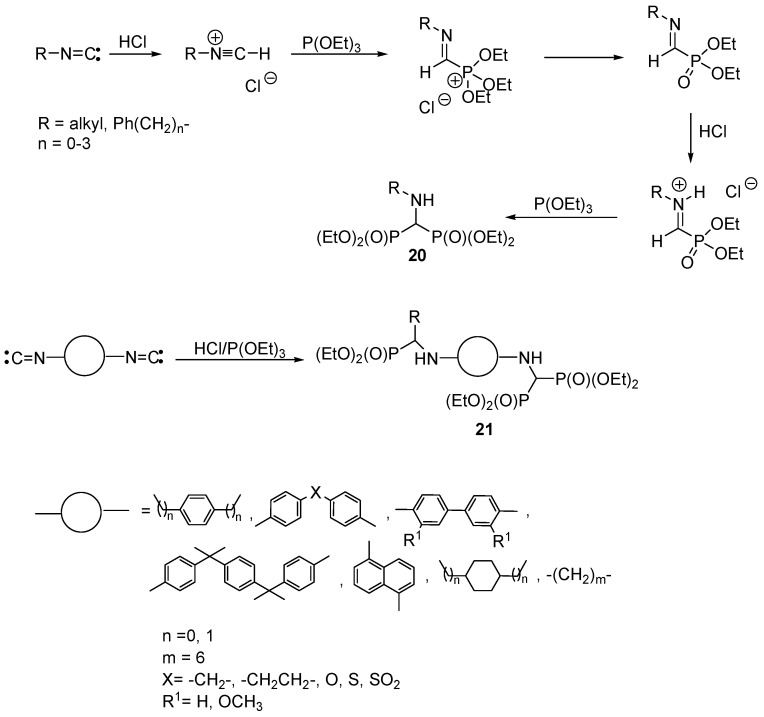
Addition of phosphites to isonitriles.

Far simpler is the addition of diethyl phosphite to isonitriles. This reaction is carried out with an excess of hydrogen chloride in aprotic solvents. Under these conditions, nitrile is converted to an onium salt, and after addition of the first phosphite molecule, iminium salt is formed, a substrate for the addition of a second phosphite molecule (Scheme 12) [53]. Using this procedure, two distinct libraries of bisphosphonates **20** and **21** have been prepared [54,55,56].

### 2.5. Synthesis via Ketophosphonates

**Scheme 13:**
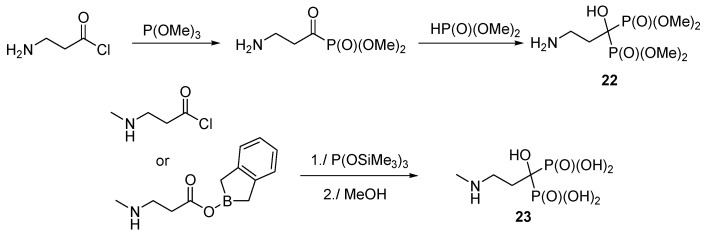
Representative syntheses via ketophosphonates.

Presumably, the addition of phosphites to ketophosphonates was the first procedure for preparation of dialkyl 1-hydroxy-1,1-bisphosphonates [16,19,57]. Starting ketophosphonates are obtained by the Arbuzov reaction of acyl chlorides with trialkyl phosphites and used as substrates in the next step, namely, the addition of dialkyl phosphite to carbonyl double bonds (a representative example for preparation of tetramethyl pamidronate **22** is shown in Scheme 13). Since ketophosphonates are unstable species [58], the desired bisphosphonates are usually obtained via one-pot procedures applying mixtures of trialkyl- and dialkylphosphonates at elevated temperature [59]. Additionally, in situ generation of dialkyl- from trialkyl phosphites is possible by the addition of a protic solvent to the reaction mixture. A useful modification of this procedure is the application of tris(trimethylsilyl) phosphite, followed by the easy removal of ester groups by methanolysis [23,60,61]. This was used to prepare a wide variety of 1-hydroxy-1,1-bisphosphonates containing amino groups [19,23,61,62,63]. Additionally, the use of bis((trimethylsilyl)oxy)phosphine generated from ammonium hypophosphite was applied to prepare hydroxybisphosphinic acids (also presented in Scheme 13) [64]. Another modification was the use of acyl phosphonamidates readily prepared from phosphoramidite type reagents and a range of acid chlorides, followed by reaction with trimethyl phosphite in the presence of pyridinium perchlorate [65].

Recently, a one-pot synthesis was described reacting carboxylic acids with catecholborane, followed by the treatment of the formed acyloxy-benzodioxaborolane with tris(trimethylsilyl)phosphite (Scheme 13) [66]. The efficiency of that simple methodology was proved by the syntheses of alendronate and *N*-methyl pamidronate (compound **23**) without additional steps for the protection/deprotection of their amine functions.

### 2.6. Three-Component Condensation of Amines with Triethyl Orthoformate and Diethylphosphite

**Scheme 14:**
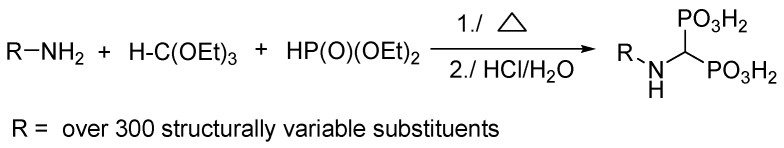
Condensation of amines with triethyl orthoformate and diethyl phosphite.

Simple three-component condensation of stoichiometric ratios of amines, diethyl phosphite and triethyl orthoformate, first reported in patent literature by Suzuki [67] and further extended by Maier [68], is perhaps the most common procedure for the preparation of a wide variety of aminomethylenebisphosphonic acids (Scheme 14). Since this reaction usually gives a complex mixture of products [56] that are difficult to separate, the resulting esters are not isolated but rather, the crude reaction mixtures are hydrolyzed, yielding bisphosphonic acids that are isolated after the hydrolytic step. Some modifications of this classic procedure have also been reported. They include the use of a solvent-free, microwave-assisted reaction [69,70,71] and reactions catalyzed by titanium dioxide [72] and by crown ethers (increasing the selectivity of the process by 10%–20%) [73]. Additionally, a reaction carried out in a micellar environment was described [74].

The three-component reaction was applied to synthesize a large series of physiologically active compounds, in some cases of very complex chemical structures, including bone antiresorptive drug candidates [75,76,77,78,79,80,81,82]; bone imaging [83,84], antiprotozoal [85,86,87,88], antibacterial [72,89,90,91,92], anti-HIV [93] and anti-inflammatory [94] agents; herbicides [95,96,97]; and complex ones for various metals [10,98].

**Scheme 15:**
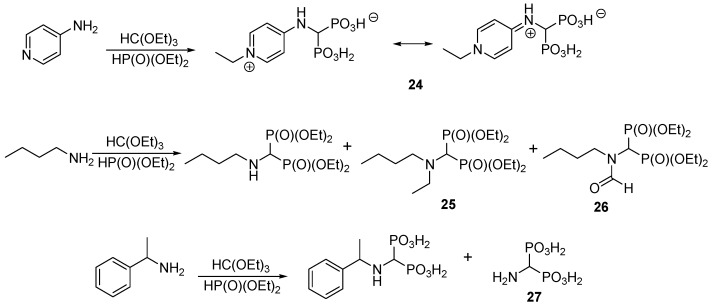
Representative side products of the three-component procedure for the synthesis of aminomethylenebisphosphonic acids.

In some cases, this reaction appears to be quite capricious and affords unexpected side-products along with the expected aminomethylenebisphosphonates (see representative examples in Scheme 15), the compositions of which are dependent on the applied conditions (molar ratio of substrates, temperature and reaction time). Most often, alkylation (for example, compounds **24** and **25**) or formylation (compound **26**) of amine moieties is observed [71,99], while in selected cases the formation of aminomethylenebisphosphonic acid (compound **27**) is observed. Because this compound was previously prepared by acid hydrolysis of *N*-benzhydrylaminomethylenebisphosphonic acid [83,100], it may be that it is formed upon hydrolysis of *N*-aryl derivatives.

**Scheme 16:**
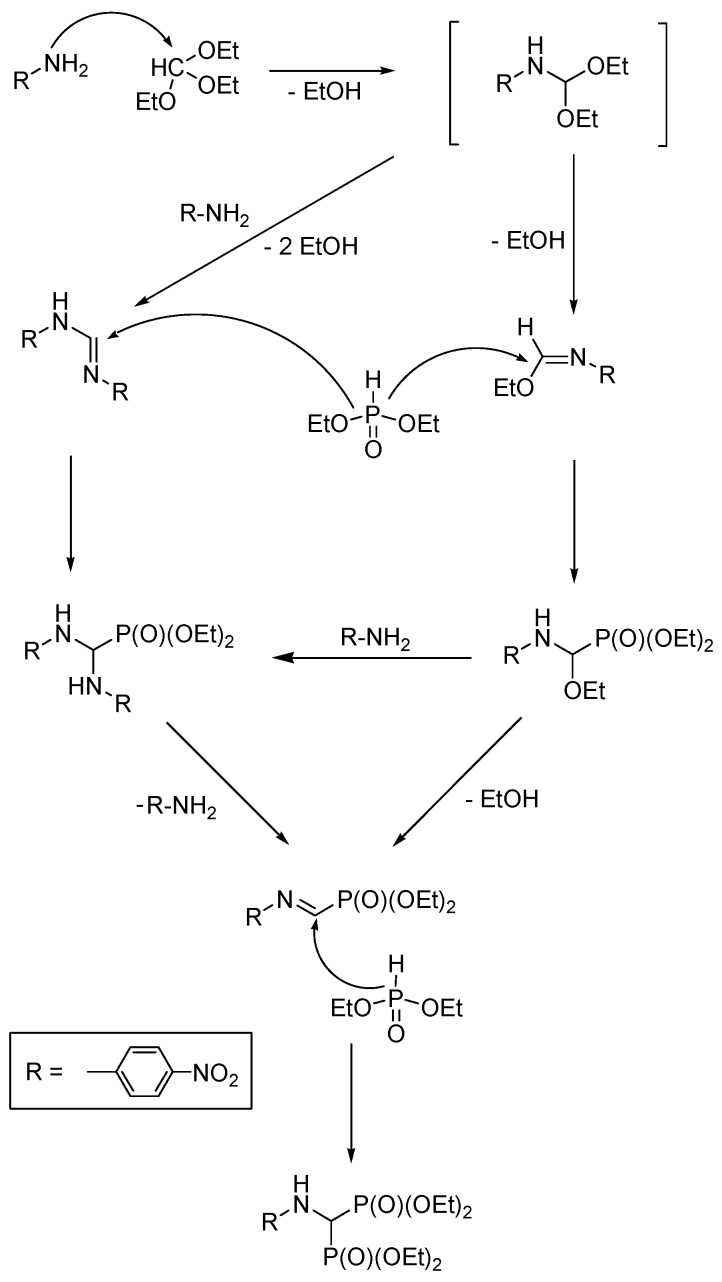
Mechanism of the three-component reaction of amines with orthoformates and phosphites.

The mechanism of this useful reaction had been thoroughly studied by using ^31^P-NMR and by isolation of all intermediates [71,72,99] and identifying interrelations between them (Scheme 16) [99]. The mechanism appeared to be quite complex because the intermediates exist in thermodynamic equilibrium. Thus, its course is strongly dependent on the properties of the used amine and applied reaction conditions.

**Scheme 17:**
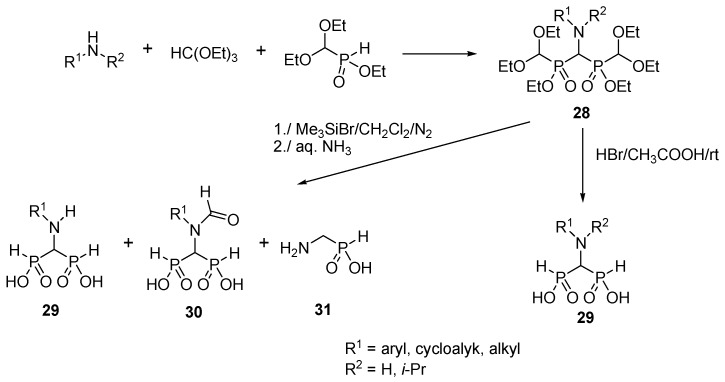
Three-component synthesis of aminobisphosphonates.

Modification of this procedure and the use of ethyl diethoxymethyl-*H*-phosphinate instead of diethyl phosphite allowed obtaining structurally variable bisphosphinates **28** (Scheme 17) [101]. Better results were obtained if the reaction was carried out under nitrogen (air oxidizes substrates and products). Notably, aromatic amines provided the desired bisphosphinates in high yields (78%–92%), whereas the reaction yields when using cyclic and aliphatic derivatives were lower. Interestingly, of many methods for the synthesis of corresponding acids **29**, acidolysis of the obtained esters **28** appeared to be optimal. The use of other conditions, including mild transesterification with trimethylsilyl bromide followed by methanolysis, resulted in the formation of side products **30** and **31**.

### 2.7. Addition of Amines to Vinylidenebisphosphonates

**Scheme 18:**
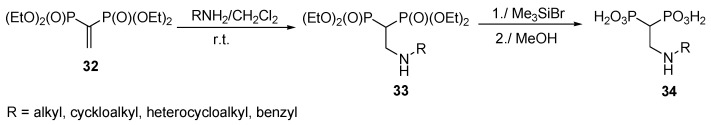
Michael addition of amines to vinylidenebisphosphonate.

Tetraethyl vinylidenebisphosphonate **32** is a versatile synthon useful for preparation of a wide variety of bisphosphonates. Its usefulness is a subject of recent comprehensive review [102]. As an electron-deficient alkene, it can undergo conjugate addition of strong and mild nucleophiles, with amines belonging to the latter class. Primary amines undergo smooth Michael addition (Scheme 18), however, the obtained compounds **33** must be quickly purified and hydrolyzed since they tend to undergo a retro-Michael reaction [103,104]. Fortunately, free acids **34** are substantially more stable.

**Scheme 19:**
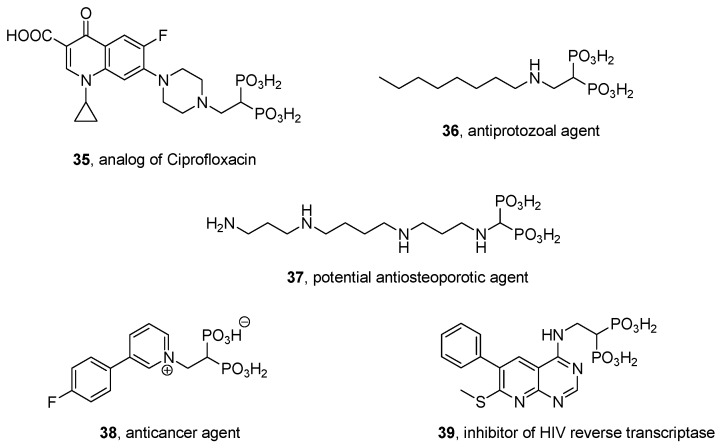
Representatives of useful bisphosphonates obtained via addition of amines to vinylidenebisphosphonate.

The reactivity of vinylidenebisphosphonates has been used for the synthesis of derivatives of various analogues of fluoroquinolone antibacterial agents [105,106], heteroaromatics with potential pharmaceutical applications [107,108], simple antiplasmodial agents [103,104], HIV reverse transcriptase inhibitors [59,93], potential anti-osteoporotic agents [109,110], and *N*-alkylated antitumor pyridines [111]. Some representatives of these compounds (**35**–**39**) are shown in Scheme 19.

**Scheme 20:**
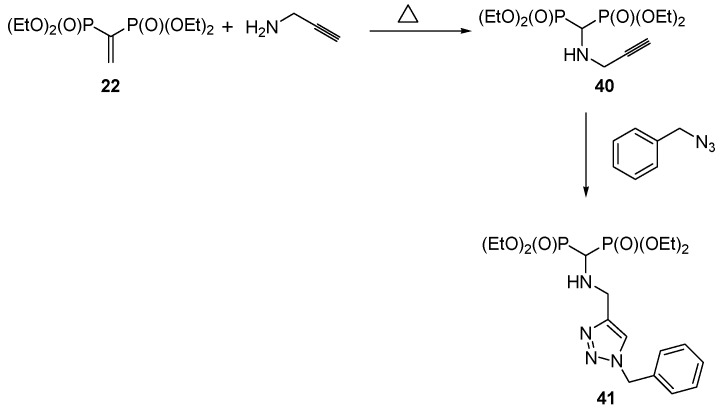
An example of the synthesis of novel bisphosphonates via “click chemistry”.

Another example, albeit far less developed, is the addition of organometallic amines to vinylidenebisphosphonate **22** (Scheme 20) [112]. Huisgen copper-catalyzed 1,3-dipolar cycloaddition of this bisphosphonate to azides was used to obtain substrate **40** for a “click reaction”. This reaction yielded compounds that may be considered aza-analogues **41** of zoledronate (Scheme 20) [113]. Similar substrates could also be obtained by addition of propargylamine to the double bond of vinylidenebisphosphonate [114].

### 2.8. Miscellaneous Procedures

The desire to obtain new aminobisphosphonate scaffolds for biological studies has stimulated numerous studies on general methods of their synthesis. The methods discussed here are mostly designed to prepare specific scaffolds or are applicable only to specific, if not unusual, substrates. Only some of them may be considered as novel, general procedures.

**Scheme 21:**
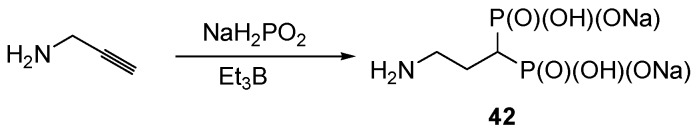
Procedure for the synthesis of bisphosphinates and their conversion into bisphosphonates.

Radical addition of sodium hypophosphite to terminal alkynes in the presence of triethylborane, which produced 1-alkyl-1,1-bis-*H*-phosphinates in moderate yields, gives access to a wide structural variety of bisphosphonates [115]. When using propargylamino acids, the corresponding bisphosphinates were obtained in satisfactory yields (a representative example is given in Scheme 21 for analogue **42** of pamidronate). Bisphosphinates are easily converted to bisphosphonates by ozonolysis.

**Scheme 22:**
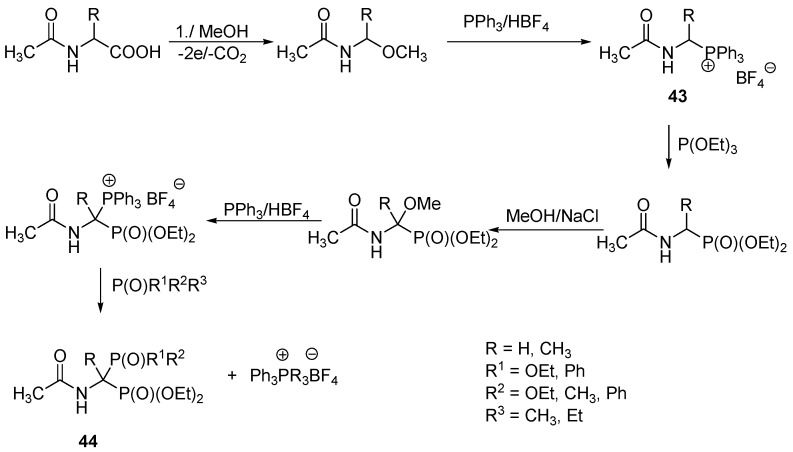
Transformation of diethyl 1-(*N*-acetylamino)-1-alkylphosphonates into bisphosphoric acid esters via the corresponding phosphonium salts.

1-(*N*-acylamino)alkylphosphonates, easily accessible from *N*-acyl-α-amino acids using a two-step transformation, underwent electrophilic activation at the α-carbon by electrochemical α-methoxylation in methanol in a process mediated by NaCl. Attempts to carry out a Michaelis-Arbuzov-like reaction of the obtained diethyl 1-(*N*-acetylamino)-1-methoxy-alkylphosphonates with triethyl phosphite failed; however, they readily reacted with triphenylphosphine (Scheme 22) [116]. The resulting diethyl 1-*(N*-acetylamino)-1-triphenylphosphoniumalkylphosphonate tetrafluoroborates **43** reacted smoothly with trialkyl phosphites, dialkyl phosphonites or alkyl phosphinites in the presence of Hünig’s base and methyltriphenylphosphonium iodide as catalysts. This gave bisphosphonates **44,** 1-phosphinylalkylphosphonates or 1-phosphinoylalkylphosphonates in good yields. This reaction has potential to become a general procedure.

**Scheme 23:**
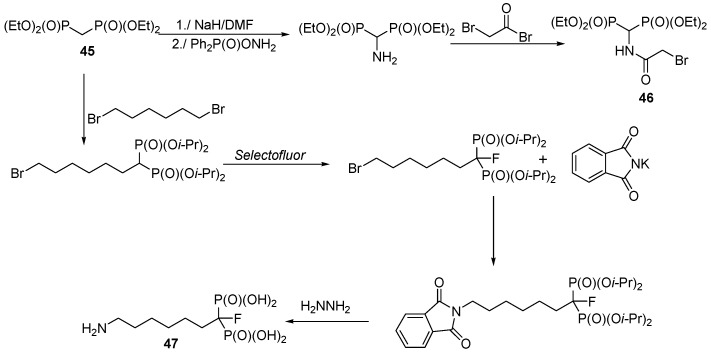
Synthesis of aminobisphosphonates via alkylation of tetraalkyl methylenebisphosphonates.

Alkylation of tetraalkyl methylenebisphosphonate **45**, a classical method for synthesizing variable bisphosphonic acids, has been rarely used for the synthesis of amino derivatives. Thus, its direct amination with the hydroxylamine ester of diphenylphosphinic acid, followed by its bromoacetylation (Scheme 23), provided substrate **46** for functionalization into glycopeptide antibiotics, in the hope that they will find an application as medication for osteomyelitis [117]. Another classical example is the monoalkylation of tetraisopropyl methylenebisphosphonate with 1,6-dibromohexane, followed by fluorination with *Selectofluor* and conversion of the remaining bromide into amine, which resulted in compound **47** (Scheme 23) [118].

**Scheme 24:**
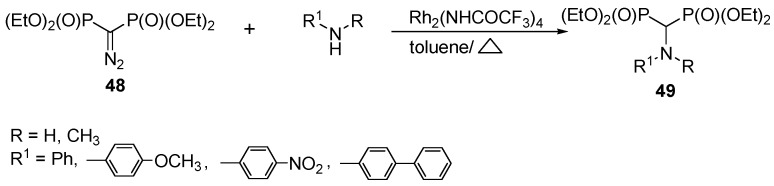
N-H insertion of the reaction of aromatic amines.

An unusual procedure is metallacarbenoid insertion of aromatic amines into tetraethyl diphosphonodiazomethane **48**, which yields corresponding aminomethylenebisphosphonates **49** (Scheme 24) [119]. Among the tested catalysts, Rh_2_(NHCOCF_3_)_4_ was found to be the best.

**Scheme 25:**
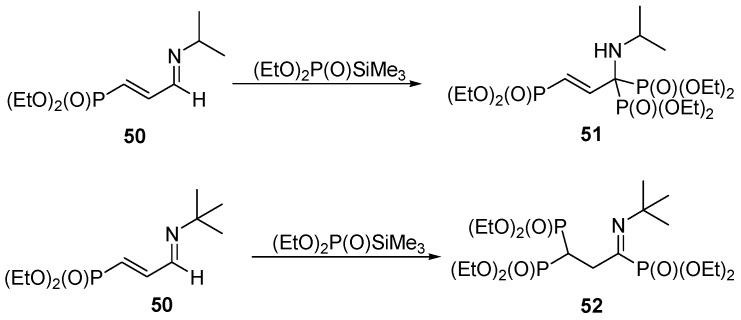
Addition of phosphites to 4-phosphono-1-aza-1,3-dienes.

A specific and unexpected reaction was the addition of silylated dialkyl phosphites to 4-phosphono-1-aza-1,3-dienes **50**, which resulted in γ-phosphono-α–aminobisphosphonates **51** (Scheme 25) [120]. This reaction is interesting in that, depending on the steric demand of the substituent present on nitrogen, double 1,2-addition or tandem 1,4-1,2-addition with formation of bisphosphonate **52** occurred (Scheme 25).

**Scheme 26:**
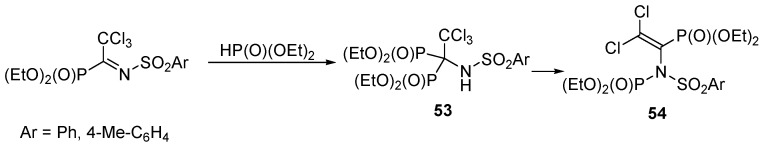
Addition of phosphites to iminophosphonates.

Additionally, the addition of phosphites to iminophosphonate esters has been studied. This reaction is limited to specific substrates, and usually the obtained bisphosphonates **53** are unstable, and phosphoryl C-N transfer to compounds **54** is observed (Scheme 26), which may be considered an example of an aza-Perkov reaction [121,122,123].

**Scheme 27:**
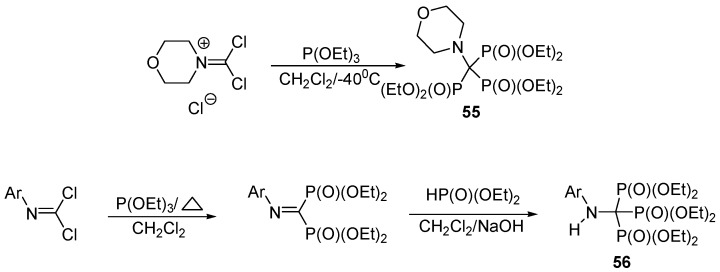
Syntheses of aminomethyltrisphosphonates.

Finally, syntheses of rare aminomethyltrisphosphonates are presented in a recent review by Romanenko and Kukhar devoted to applications of methylidynetrisphosphonates [124]. Two procedures for the synthesis of bis- and trisphosphonates **55** and **56** taken from this review and described in patent literature are depicted in Scheme 27.

## 3. Functionalization of Aminobisphosphonates

Bisphosphonates are known for their affinity to bone tissue, and thus their conjugation to various drugs has been quite intensively studied; they are expected to serve as system-targeting drugs [6]. Because dronic acids are produced at industrial scale and are thus readily available and inexpensive, they are most frequently used for that purpose. However, their extreme hydrophilic character means that they are practically insoluble in most organic solvents, which limits the use of aqueous media or requires their conversion into phosphonate esters prior to functionalization. Unfortunately, the methods for direct esterification of phosphonate moieties are scarce and usually give unsatisfactory results; these esters must generally be synthesized independently.

Methods for functionalizing aminobisphosphonic acids with structurally variable molecules were recently reviewed [12]; therefore, in this review, the methods arbitrarily chosen as the most representative are reported. 

### 3.1. Direct Acylation of Aminobisphosphonic Acids

**Scheme 28:**
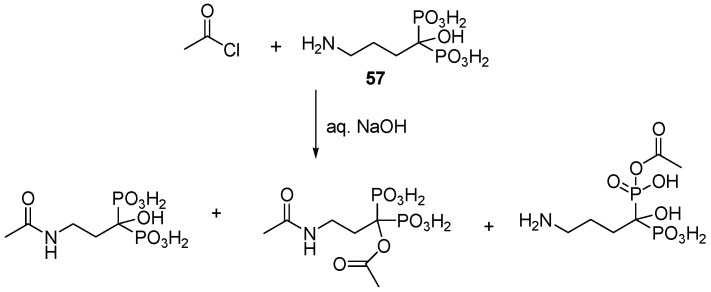
Direct acylation of alendronic acid with indication of possible side-products.

Direct acylation of aminobisphosphonic acids is difficult because this reaction is accompanied by the possible competitive acylation of phosphonic groups [125,126] and hydroxylic groups when amino-1-hydroxy-1,1-bisphosphonic acids are substrates [126,127]. A representative example of this reaction is given in Scheme 28 for alendronic acid **57**.

**Scheme 29:**
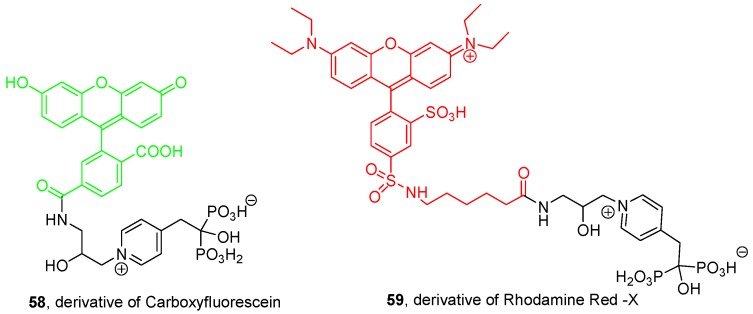
Representative fluorescent imaging probes obtained via acylation of bisphosphonic acids (green—carboxyfluoresceine fragment, red—rhodamine Red-X fragment).

Acylation of dronic acid salts with acyl chlorides in sodium hydroxide in water or in water/propanol solutions is the simple Schotten-Baumann variant [127,128]. Acidification of the solution results in the precipitation of the desired acids or their monosodium salts. To conjugate aminobisphosphonic acids with molecules bearing carboxylic groups, classical coupling protocols used in peptide synthesis have also been used. These include the activation of carboxylic groups with *N*,*N*’-dicyclohexylcarbodiimide (DCC) [129,130]; the use of previously prepared succinimidate esters to obtain 21 extremely complex fluorescent imaging probes (representative examples of a green fluorescent dye **58** and a red fluorescent dye **59** are shown in Scheme 29) [131]; and *N*,*N*’-dicarbonylimidazole, which was used to conjugate pamidronate to pullan (polysaccharide composed of maltotriose units) [132] to obtain a system for bone regeneration.

Acylation of aminobisphosphonic acids with small acids, such as acrylic acid [133], chloroacetic acid [134] or succinic acid [135], provided useful substrates for further functionalization of larger molecules, such as macrocycles devised for lanthanide ion complexation, chitosan, and hyaluronan.

### 3.2. Direct Acylation of Tetraethyl Aminobisphosphonates

**Scheme 30:**
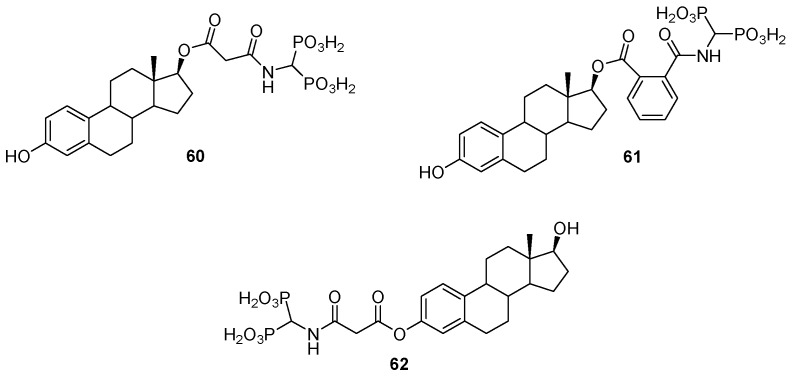
Representative structures of bone-specific estrogens.

Esters are far more suitable substrates for acylation than free phosphonic acids, with tetraethyl aminomethylenebisphosphonate being the most popular substrate. It has been used to obtain structurally variable conjugates with estradiol (compounds **60**, **61** and **62** in Scheme 30) using classical peptide synthesis coupling agents such as DCC and DPPA (diphenylphosphoryl azide) [136]. These conjugates were synthesized as bone-specific estrogens in the hope that they will protect elderly women from bone loss resulting from osteoporosis.

A derivative of raloxifene, a selective estrogen receptor or modulator, was obtained using a suitable acid chloride as substrate [137], whereas radioligands, which are selectively bound to bone tissue, have been synthesized by using DCC/HOBt (hydroxybenzotriazol) activation [138] and antibacterial bisphosphonated benzoxazinorifamycin prodrugs using EDCl (1-ethyl-3-(3-dimethylaminopropyl)carbodiimide) [139].

In this case, acylation of aminobisphosphonic acids with small acids was also applied as a starting step to produce antibacterial agents against osteomyelitis [88] and bone imaging macrocycles [81], using acid chlorides as acylating agents.

Using DCC as a coupling agent, diethyl 1-(2-aminoethylamino)-1,1-ethylbisphosphonate was acylated by structurally variable natural acids, such as folic acid [140,141], ursulonic and betulinic acids [142], and trolox [141,143].

**Scheme 31:**
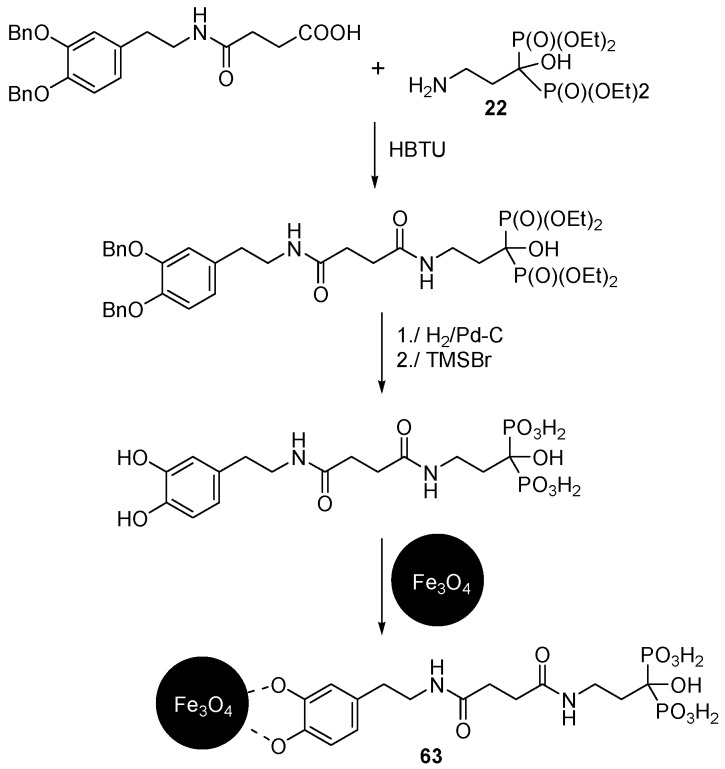
Synthesis of bisphosphonate functionalized magnetic nanoparticles.

Acylation of pamidronic acid ester **22** by using HBTU (2-(1*H*-benzotriazol-1-yl)-1,1,3,3-tetramethyluronium hexafluorophosphate) was used to prepare a catecholic derivative, which was then bound to the surface of magnetic iron oxide nanoparticles (Scheme 31) [144]. The particle **63** has been designed to remove uranyl ions from blood.

### 3.3. Synthesis of Building Blocks for Polymer Chemistry

**Scheme 32:**
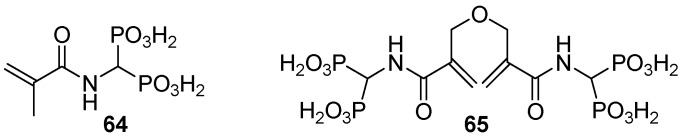
Bisphosphonylated methacrylamide monomers.

Self-etching adhesives are polymeric materials containing phosphonate groups that have become popular in restorative dentistry because they allow strong bonds between dental hard tissues (enamel and dentin). One possibility for their preparation is to obtain monomers containing phosphonic groups [145]. Such methacrylamide monomers **64** and **65** were synthesized by acylation with suitable acryloylchlorides (Scheme 32) [146]. Studies of their photopolymerization indicated that they may be suitable for potential use in dentistry.

A similar monomer, acryloylated pamidronate, has been used to obtain a hyaluronic acid derivative that is dually polymerized with cross-linkable hydrazide groups and bisphosphonate ligands. By mixing bisphosphonate polymer with calcium ions and aldehyde-derivatized hyaluronic acid, a hybrid hydrogel was obtained, which quickly mineralizes [147]. Such a system is of interest as a mediator for fast bone regeneration.

**Scheme 33:**
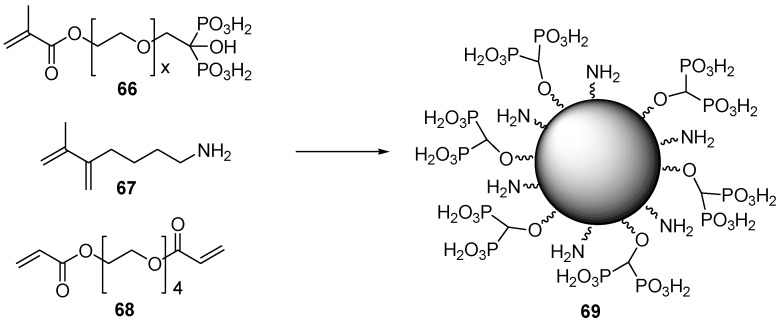
Synthesis of polymeric nanoparticles as potential dye and drug carriers.

By dispersion copolymerization of three monomers (methacrylate bisphosphonate **66**, *N*-(3-aminopropyl) methacrylamide **67**, and tetra(ethylene glycol) diacrylate **68**) (Scheme 33), polymeric nanoparticles **69** were obtained [148]. Their size distribution was controlled by changing various polymerization parameters. By covalent attachment of a drug and/or a dye to amino groups (such as a near IR fluorescent dye), a theranostic system may be obtained.

Macromolecular co-conjugates of bisphosphonate and ferrocene were synthesized by means of Michael addition copolymerization of methylenebisacrylamide (MBA) with primary amines-6-amino-1-hydroxyhexylidene-1,1-bisphosphonate and 4-ferrocenyl-butamidopropylamine [149]. The mass percentage incorporation of ferrocene analogues was found to be between 4%–5%, and 10%–12% for bisphosphonate. Such polymers could be selectively bound to bone tissue and slowly release anticancer ferrocene derivatives at this target site.

### 3.4. Miscellaneous

Other means to functionalize amino moieties are quite scarce and dispersed. Classical ones include the formation of Schiff bases, which are then alkylated [150] or reduced [126], and synthesis of thioureido derivatives as intermediates in the preparation of heterocyclic compounds [151,152,153].

**Scheme 34:**
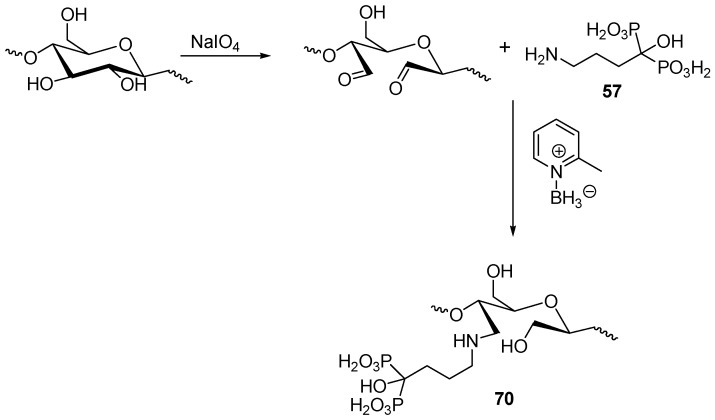
Periodate oxidation of cellulose followed by reductive amination with sodium alendronate.

The functionalization of polysaccharides is the gateway of aminobisphosphonates into nanoscience. For example, phosphonated cellulose was utilized to obtain nanocellulose with good thermal stability and potential intumescent properties. It was synthesized from birch pulp via sequential periodate oxidation and reductive amination using alendronate **57** as a phosphonating reagent (Scheme 34) [154]. After high-pressure homogenization, bisphosphonate cellulose nanofibers or nanocrystals of the general formula **70** were obtained, depending on the initial oxidation degree.

Alendronate was also bound to conjugates of pullulan and paclitaxel, which were bound to the polysaccharide by a cathepsin K-sensitive tetrapeptide spacer. This should ensure release of paclitaxel in bones. Then, bisphosphonate was covalently conjugated to the sugar chain via a polyethyleneglycol chain, using a technique similar to that described above (identical to those shown in Scheme 34) [155]. This system exhibited strong antiproliferative action against several cancer cell lines.

## 4. Conclusions

Aminobisphosphonic acids are gaining significant interest as a class of compounds with promising physiologic activity, with some representatives already commercialized as bone resorption inhibitors, and therefore useful drugs against osteoporosis and related bone disorders. This leads to both the modification of existing and the elaboration of novel procedures for their preparation. There are several commonly used reactions for this purpose; however, they have also been modified in the last decade to be tailored to specific biological needs. A few novel procedures have also been developed. Functionalization of simple aminobisphosphonic acids is a difficult task because of their strongly polar character. However, successful examples of functionalization of the amino moieties of these compounds have provided promising diagnostics and novel therapeutic agents.
